# Synbiotic effects of β-glucans from cauliflower mushroom and *Lactobacillus fermentum* on metabolic changes and gut microbiome in estrogen-deficient rats

**DOI:** 10.1186/s12263-017-0585-z

**Published:** 2017-11-09

**Authors:** Seong-Yeop Jeong, Suna Kang, Cao Shi Hua, Zhang Ting, Sunmin Park

**Affiliations:** 1 Department of R&D, Microbial Institute for Fermentation Industry, Sunchang, South Korea; 2 Department of Food & Nutrition, Obesity/Diabetes Center, Hoseo University, 165 Sechul-Ri, BaeBang-Yup, Asan-Si, ChungNam-Do 336-795 South Korea

**Keywords:** Cauliflower mushroom, β-Glucan, *Lactobacillus fermentum*, Gut microbiota, Ovariectomy, Synbiotics

## Abstract

**Background:**

We investigated whether the long-term consumption of a symbiotic formulation with *Lactobacillus fermentum* (probiotic) and β-glucan from cauliflower mushroom (prebiotic) would delay the progression of post-menopausal symptoms in ovariectomized (OVX) rats and explored their mechanisms of action, including changes in gut microbiota.

**Methods:**

OVX rats were fed with high-fat diets containing 1% dextrin (control), 1% lyophilized cauliflower mushroom extract (CFM), 0.1% *L*. *fermentum* JS (LFE), 1% CFM plus 0.1% LFE (CFLF), or 30 μg 17β-estradiol/kg body weight (positive-control) for 8 weeks.

**Results:**

CFM contained 95.8% β-glucans. OVX increased the ratio of *Firmicutes* and *Bacteroidetes* in the large intestines. Only CFLF lowered tail skin temperature without increasing serum 17β-estradiol and uterine index. Visceral fat mass was lower in CFLF and positive-control groups by increasing daily energy expenditure and fat oxidation. Dyslipidemia induced by OVX was improved by CFM and CFLF as much as in the positive-control group. Homeostasis model assessment estimate of insulin resistance was lower in CFLF than in the positive-control. Hepatic insulin signaling (pAkt➔GSK-3β) was potentiated in the ascending order of the control, LFE, CFM, CFLF, and positive-control. AMPK phosphorylation showed similar patterns of hepatic insulin signaling but LFE increased it more than CFM. The changes in gut microbiota were prevented by CFLF in OVX rats, and the ratio of *Firmicutes* and *Bacteroidetes* in the CFLF was similar to the positive-control group.

**Conclusion:**

OVX changed gut microbiota and was associated with menopausal symptoms; however, the synbiotics, CFM and LFE, prevented menopausal symptoms and improved the gut microbiota in estrogen-deficient rats.

## Background

Estrogen deficiency causes disturbances of energy, lipid, glucose, and bone metabolism and induces vasomotor symptoms such as daytime hot flushes, night sweats, sleep and mood disorder, and difficulty in concentration. These menopausal symptoms result in a lower quality of life and increase the incidence of metabolic disease. Although hormone replacement therapy ultimately prevents menopausal symptoms and improves quality of life, hormone replacement therapy is often not recommended due to its risk of adverse side-effects [1]. Alternative therapies with less adverse effects have been suggested for the treatment of menopausal symptoms.

*Firmicutes* among the gut microbiota is associated with obesity and its progression towards metabolic disease [2], and dietary interventions to modulate the gut microbiota can alleviate metabolic diseases [3]. Sex hormones including estrogen and testosterone have been reported to modulate microbial communities [4], and gut microbiota influence systemic levels of sex hormones [5, 6]. Intestinal tissues mainly contain estrogen receptor (ER)-β. ER-β affects the composition of gut microbiota in female mice [7]. Cox-York et al. [8] have demonstrated that ovariectomized (OVX) rats with higher aerobic capacity increase microbial diversity and the number of the *Bacteroidetes phylum* in the gut. In addition, equol production in the gut produces the beneficial effects of soy and isoflavones on menopausal symptoms and gut microbiota is associated with equol production in the gut from soy-isoflavone concentrates in post-menopausal women [9]. Thus, the changes of gut microbiota composition can prevent the menopausal symptoms.

Diets including prebiotics and probiotics can modulate gut microbiota [7, 10]. Probiotics consumption seems to be a feasible approach to modulate the intestinal microbiota and to maintain or restore human health [11]. *Bifidobacterium* and *Lactobacillus*, natural components of the colon microbiota, are the most commonly used probiotics in many functional foods and dietary supplements. Various *Lactobacillus* species such as *Lactobacillus fermentum*, *Lactobacillus plantarium*, and *Lactobacillus reuteri* are naturally present in fermented foods and milk products [12] and especially, *L. fermentum*, which was isolated from human milk, can inhibit pathogens, decrease cholesterol synthesis, and change gut microbiota [13, 14]. Dietary supplementation with prebiotics, including lactulose, galacto-oligosaccharides, and fructo-oligosaccharides, increases the contents and proportion of bifidobacteria in the intestines [15] and improves nutrient absorption, prevents gut inflammation, and stimulates energy and glucose metabolism [16, 17]. Synbiotics, a combination of probiotics and prebiotics, may have better efficacy for changing gut microbiota and to improving metabolic symptoms [17]. However, there are still some debates about consumption of prebiotics and probiotics, gut microbiota, and metabolic efficacy.

Cauliflower mushroom (*Sparassis crispa* Wulf.:Fr.) contains about 40% of six-branched 1,3-beta-glucan that can change gut microbiota [18, 19]. However, β-glucan is known to improve inflammation and to alleviate inflammatory bowel diseases, cancer, atopic dermatitis, and arthritis. In addition, β-glucan potentiates glucagon-like peptide-1 secretion in L cells in the intestines to improve glucose metabolism. Thus, cauliflower mushroom may prevent the energy, glucose, lipid, and bone metabolism disturbances that occur in estrogen-deficient animals while modulating the gut microbiota.

We hypothesized that the long-term consumption of *L*. *fermentum* (probiotics) and cauliflower mushroom (prebiotics) would prevent and/or delay the progression of post-menopausal symptoms in estrogen-deficient animals with diet-induced obesity. The present study tested this hypothesis and explored their mechanisms of action in OVX rats fed a high-fat diet supplemented with *L*. *fermentum* and cauliflower mushroom.

## Methods

### Water extract of cauliflower mushroom and β-glucan contents

Cauliflower mushroom was purchased from Haemaru Biotechnology (Asan, Korea). Dried cauliflower mushroom was powdered, and the powder was extracted with water at 90 °C for 4 h in an extractor. The extract was concentrated by 50% with a rotary evaporator and centrifuged at 8000×*g* for 30 min. The concentrates were then freeze dried, and the yield of the mushroom was 28.8%.

To calculate the β-glucan contents, cauliflower mushroom extract was sequentially degraded with digestive enzymes by incubating in a shaking incubate with specific pH and temperature for 2 h. The enzymes used to digest the cauliflower mushroom were amylase (20 units, pH 6.9) at 20 °C, cellulase (50 units, pH 5.0) at 37 °C, protease (10 units, pH 7.5) at 37 °C, and amyloglucosidase (70 units, pH 4.8) at 60 °C. After digesting the cauliflower mushroom with enzymes, 95% ethanol was added into the digested mixture and the mixture was left at 4 °C for 12 h. The mixture was centrifuged at 10,000 rpm for 10 min, and the precipitates were mixed with water. The mixture was homogenized and diluted with distilled water, and then sulfuric acid was added (1:5). The mixture was left at room temperature for 20 min, and the optical density was measured at 470 nm. Glucose solution was used as a standard.

### Ovariectomy operation

Female Sprague–Dawley rats (weighing 271 ± 9 g) were housed individually in cages in a controlled environment (23 °C, with a 12-h light/dark cycle). Animal care and surgery were conducted according to the NIH Guide for the Care and Use of Laboratory Animals. The Sprague–Dawley rats from DBL (Yeumsung-Kun, Korea) had an acclimation for 1 week in our animal facility. The rats had an ovariectomy (OVX) operation under anesthesia by a subcutaneous injection of a mixture of ketamine and xylazine (100 and 10 mg/kg body weight, respectively) [20]. A mid-ventral incision was made, and both ovaries were dissected after each ovary was separated by the ligation of the most proximal portion. The OVX rats were randomly assigned into five groups of 10 each.

*L*. *fermentum* JS is an acid and bile salt-resistant lactic acid bacteria [21]. *L*. *fermentum* JS (KCCM 10499) has been registered as a probiotic for improving intestinal health and immunity [22, 23] in Korea Food & Drug Administration. *L*. *fermentum* JS KCCM 10499 was routinely grown in Difco MRS broth or agar (BD, NJ, USA) and incubated in shaking incubator (Daihan Science, Wonjoo, Korea) with aerobic condition at 37 °C for 24 h. After incubation, the media was centrifuged at 900×*g* and the precipitates were lyophilized. The freeze-dried powder contained 1 × 10^8^–1 × 10^10^ CFU *L. fermentum* JS/g.

### Diet preparation

High-fat diets were used in the study since they are known to exacerbate the progression of menopausal symptoms compared with a low fat diet [24–26]. Water extracts of cauliflower mushroom and *L*. *fermentum* JS KCCM 10499 were purchased from Haemaru-Biotech (Cheonan, Korea) and Well-being LS (Gang Reung, Korea), respectively, and were supplemented into high-fat diets. The high-fat diet was a semi-purified modified AIN-93 formulation containing 37 energy percent (En%) from carbohydrates, 20 En% from protein, and 43 En% from fats [27]. The sources of carbohydrate, protein, and fat were starch plus sugar, casein (milk protein), and lard (CJ Co., Seoul, Korea), respectively. CFM included 1% lyophilized water extracts of cauliflower mushroom plus 0.1% casein; LFE contained 0.1% *L*. *fermentum* (1 × 10^8^–1 × 10^10^ CFU/g) plus 1% dextrin; CFLF included 1% CFM plus 0.1% LFE; control contained 1% dextrin plus 0.1% casein; or positive-control contained 30 μg/kg body weight 17β-estradiol plus1% dextrin and 0.1% casein. The CFM and LFE powders were homogeneously mixed with the vitamin and mineral mixture and sugar components of the diet. The mixture was sifted to remove the lumps. Each mixture was blended with the appropriate amounts of starch, casein, and lard, sifted again, and stored at 4 °C. The nutrient composition of the diets was the same. The amounts of each supplement consumed were calculated from daily food intake.

### Experimental design

Sixty OVX rats were randomly assigned to the following groups: (1) CFM, (2) LFE, (3) CFLF, (4) positive-control (30 μg/kg body weight 17β-estradiol), or (5) OVX-control (dextrin; placebo). The OVX rats had free access to water and their respective diets for 8 weeks. After an overnight-fast, the serum glucose levels, food intake, and body weights were measured every Tuesday at 10 am.

### Tail skin temperature

The tail skin temperature was measured during the dark-period every week of the experimental period using an infrared thermometer for small rodents (BIO-152-IRB, Bioseb, Chaville, France) [26]. Three measurements were made 10 min apart each time, and the average value was used as a single data point.

### Body composition measurement

At the seventh week of the experimental period, the body composition of the rats was measured in a dual-energy X-ray absorptiometer (DEXA; Norland pDEXA Sabre; Norland Medical Systems Inc., Fort Atkinson, WI, USA) after calibrating it with a phantom supplied by the manufacturer. After anesthetization with ketamine and xylazine (100 and 10 mg/kg bw, respectively), bone mineral density (BMD) and lean body mass were assessed in the leg and hip using the DEXA instrument equipped with the appropriate software for small animals [26]. Fat mass was measured in the leg and abdominal areas using the DEXA equipment.

### Energy expenditure analysis by indirect calorimetry

At 2 days after the DEXA analysis, energy expenditure was assessed at the beginning of the dark-phase of the light/dark cycle after removing foods for 6 h. The rats were acclimated into the metabolic chambers (airflow = 800 mL/min) for 30 min, and then the chambers were connected with a computer-controlled O_2_ and CO_2_ measurement system (BIOPAC Systems, Inc., Goleta, CA). Oxygen consumption (VO_2_) and carbon dioxide production (VCO_2_) were measured over 1 min intervals and integrated over periods of 30 min. The VO_2_ and VCO_2_ values were corrected for the animals’ metabolic body size (kg^0.75^). The respiratory quotient and resting energy expenditure were calculated using reported equations [20, 26]. Carbohydrate and fat oxidation were calculated from non-protein oxygen consumption according to their relative oxidative proportions. They were reported as the amount of oxygen consumed per gram of substrate oxidized [24, 26].

### Glucose homeostasis, lipid profiles, and sample collection at the end of experiment

An oral glucose tolerance test (OGTT) was performed on overnight-fasted animals at the eighth week. Blood samples were taken by tail bleeding at 0, 10, 20, 30, 40, 50, 60, 70, 80, 90, and 120 min after orally giving 2 g of glucose/kg body weight. The serum glucose levels were measured with a Glucose Analyzer II (Beckman, Palo Alto, CA). The serum insulin levels were determined at 0, 20, 40, 90, and 120 min using a radioimmunoassay kit (Linco Research, Billerica, MA). The areas under the curves (AUC) of the first (0–40 min) and second (40–120 min) parts for the serum glucose and insulin levels were calculated using the trapezoidal rule.

Three days after the OGTT, food was removed for 6 h and an intraperitoneal insulin tolerance test (IPITT) was conducted. The serum glucose levels were measured every 15 min for 90 mins after an intraperitoneal injection of insulin (0.75 U/kg body weight). At 2 days after the IPITT, the rats were anesthetized with ketamine and xylazine (100 and 10 mg/kg body weight, respectively) and human insulin (5 U/kg body weight) was injected through the inferior vena cava after collecting blood by abdominal cardiac puncture. The epididymal and retroperitoneal fat mass and uteri were then collected and weighed. The uterus index was calculated as the uterus weight divided by the body weight. Feces in the large intestine were collected from the large intestine of all rats in each group. Feces from two of samples were pooled to make five fece samples of each group. The pooled contents were mixed, and the gut microbiomes were measured. Serum, tissue, and large intestine specimen were stored at − 70 °C for biochemical analysis.

Insulin resistance and insulin secretion capacity were estimated using the homeostasis model assessment estimate (HOMA) of insulin resistance (HOMA-IR) and insulin secretion (HOMA-B). HOMA-IR and HOMA-B were calculated as previously described [26, 28]. Serum 17β-estradiol levels were assessed by ELISA kits (Enzo Life Sciences, NY, USA). The levels of serum tumor necrosis factor (TNF)-α and monocyte chemoattractant protein-1 (MCP-1) were measured with ELISA kits (R & D Systems, Minneapolis, MN). Total cholesterol, HDL cholesterol, and triglycerides levels in the circulation were assessed by using colorimetry kits for (Asan Pharmaceutical, Seoul, Korea). Serum LDL cholesterol was calculated with the Friedewald equation [29].

### Gut microbiome

Gut microbiome was measured from five fece samples from each group by Microgen (Seoul, Korea) by analyzing metagenome sequencing using next-generating sequencing. Bacterial DNA was extracted from each sample by Power water DNA Isolation Kit (MoBio, Carlsbad, CA) according to the manufacturer’s instructions. Each library was prepared using PCR products according to the GS FLX plus library prep guide. Libraries were quantified using Picogreen assay (Victor 3). The emPCR, corresponding to clonal amplification of the purified library, was carried out using the GS-FLX plus emPCR Kit (454 Life Sciences, Branford, CT). Briefly, libraries were immobilized onto DNA capture beads. The library beads were added to amplification mix and oil, and the mixture was vigorously shaken on a Tissue Lyser II (Qiagen, Valencia, CA) to create “micro-reactors” containing both amplification mix and a single bead. Emulsion was dispensed into a 96-well plate and the PCR amplification program was run according to the manufacturer’s recommendations. The 16S universal primers, 27F (5′ GAGTTTGATCMTGGCTCAG 3′) and 518R (5′ WTTACCGCGGCTGCTGG 3′), were used for the amplification of 16 s rRNA genes by PCR. PCR was conducted in the FastStart High Fidelity PCR System (Roche, Basel, Switzerland) under the following conditions: 94 °C for 3 min followed by 35 cycles of 94 °C for 15 s, 55 °C for 45 s and 72 °C for 1 min, and a final elongation step at 72 °C for 8 min. After the PCR procedure, we purified the products using AMPure beads (Beckman coulter, Brea, CA).

### Next-generation sequencing using Roche 454 GS-FLX plus

Sequencing was performed by the Macrogen Ltd. (Seoul, Korea). Following PCR amplification, the emulsion was chemically broken and the beads carrying the amplified DNA library were recovered and washed by filtration. Positive beads were purified using the biotinylated primer A (complementary to adaptor A), which binds to streptavidin-coated magnetic beads. The DNA library beads were then separated from the magnetic beads by melting the double-stranded amplification products, leaving a population of bead-bound single-stranded template DNA fragments. The sequencing primer was then annealed to the amplified single-stranded DNA. Lastly, beads carrying amplified single-stranded DNA were counted with a Particle Counter (Beckman Coulter). Sequencing was performed on a Genome Sequencer FLX plus (454 Life Sciences), and each sample was loaded in one region of a 70–75-mm PicoTiter plate (454 Life Sciences) fitted with an eight-lane gasket.

### Immunoblot analysis

The liver was lysed with a 20 mM Tris buffer (pH 7.4) containing 2 mM EGTA, 137 mM NaCl, 1% NP40, 10% glycerol, and 12 mM α-glycerol phosphate and protease inhibitors. Lysates containing equal amounts of protein (30–50 μg) were used for immunoblotting with specific antibodies against protein kinase B (PKB/Akt), glycogen synthase (GSK)-3β, AMP kinase (AMPK), phosphoenol-pyruvate carboxykinase (PEPCK), and β-actin, and phosphorylated forms of PKB^ser473^, GSK-3β ^ser9^, and AMPK^the172^ (Cell Signaling, Danvers, MA), as previously described [24]. The intensity of protein expression was determined using Imagequant TL (Amersham Biosciences, Piscataway, NJ).

### Statistical analysis

Statistical analysis was performed using SAS software version 7 (SAS Institute). Sample size was calculated using a G power program (power = 0.90), and sample size of each group was 10. Results were expressed as means ± standard deviation (SD) when the normal distribution was checked using Proc univariate. The variables that were measured over multiple time points were tested with two-way repeated measure ANOVA, with time and group as the independent variables and an interaction term between time and groups. One-way ANOVA was used to determine the metabolic effects of the OVX-control, CFM, LFE, CFLF, and 17β-estradiol (a positive-control) groups at a single time point, when the results were measured once at the end of the experiment. Significant differences in the main effects among the groups were identified by Tukey’s test at *p* < 0.05.

## Results

### β-Glucan contents of CFM and *L*. *fermentum* in LFE

The β-glucan contents were 33.4 ± 2.2 g/100 g cauliflower mushroom. The water extracts mainly contained β-glucans (about 95.8%). The yield of its water extracts was 28.8% because some β-glucan was lost during the water extraction process. Rats in the CFM group consumed 0.15–0.2 g β-glucan and those in the LFE group had 1–2 × 10^7^ CFU on a daily basis. Rats in the CFLF group consumed 0.15–0.2 g β-glucan plus 1–2 × 10^7^ *L*. *fermentum* daily.

### Serum 17β-estradiol levels, uterine index, tail skin temperature and inflammation index

Serum 17β-estradiol levels were much lower in the OVX-control group than the positive-control group and the levels were slightly higher in CFLF, but it was not significantly different (Table 1). Uterine index was also much lower in the OVX-control group than the positive-control group, and none of the treatments changed the index. Due to the estrogen deficiency, tail skin temperature was higher in OVX-control group than the positive-control (Table 1). Only CFLF lowered skin temperature.

**Table 1:** Serum 17β-estradiol levels, uterine index, and skin tail temperature

	Control	CFM	LFE	CFLF	Positive-control
Serum 17β-estradiol levels (pg/mL)	1.6 ± 0.6^b^	1.9 ± 0.7^b^	1.7 ± 0.5^b^	2.0 ± 0.7^b^	7.3 ± 1.1^a^
Uterine index	0.49 ± 0.10^b^	0.52 ± 0.11^b^	0.50 ± 0.12^b^	0.56 ± 0.10^b^	1.80 ± 0.29^a^
Tail skin temperature (°C)	29.6 ± 0.17^a^	28.8 ± 0.17^ab^	29.3 ± 0.15^a^	28.2 ± 0.14^b^	27.9 ± 0.15^b^
Serum TNF-α (pg/mL)	52.1 ± 4.6^a^	46.2 ± 4.5^b^	48.5 ± 4.4^ab^	41.1 ± 4.2^c^	42.5 ± 4.3^bc^
Serum MCP-1 (pg/mL)	60.5 ± 6.4^a^	50.4 ± 5.7^b^	55.3 ± 5.6^b^	43.7 ± 4.9^c^	44.5 ± 4.7^c^

Values represented means ± standard deviation (*n* = 10)

Different letters represent a significant difference in Tukey test at *p* < 0.05

*CFM* 1% water extract of cauliflower mushroom, *LFE* 0.1% *L*. *fermentum*, *CFLF* 1% water extract of cauliflower mushroom +0.1% *L*. *fermentum*, *positive-control* 30 μg/kg body weight 17β-estradiol, *TNF-α* tumor necrosis factor-a, *MCP-1* monocyte chemoattractant protein-1

Serum TNF-α and MCP-1 levels, inflammation indexes, were higher in the control group than the positive-control group. They were lower in both CFM and LFE than the control group, and CFLF synergistically decreased then to similar levels as the positive-control group (Table 1).

### Energy metabolism

The OVX-control group had higher body weights than the positive-control group and CFLF prevented its increase in OVX rats (Table 2). As a result, final body weight was greater in the OVX-control group than the positive-control group but CFLF also lowered final body weight but it was not significantly different from the OVX-control or positive-control groups (Table 2). Peri-uterine fat and retroperitoneum fat mass, the visceral fat mass, was much greater in OVX-control group than the positive-control group. CFLF lowered the visceral fat mass to less than the OVX-control, but the visceral fat mass in the CFLF was greater than that in the positive-control group (Table 2). The body weight gain and visceral fat mass are associated with the balance of food intake and energy expenditure. Food intake was not significantly different among any of the groups (Table 2). However, OVX-control rats exhibited a decrease in daily energy expenditure compared to the positive-control and CFLE prevented its decrease in OVX rats. Carbohydrate oxidation as an energy fuel was not significantly different among all the groups. However, fat oxidation was much lower in the OVX-control group than the positive-control group and CFLF protected its decrease in OVX rats (Table 2).

**Table 2:** Body composition and energy metabolism at the end of experimental periods

	Control	CFM	LFE	CFLF	Positive-control
Final weight (g)	416 ± 29^a^	407 ± 38^a^	414 ± 42^a^	395 ± 19^ab^	373 ± 32^b^
Weight gain (g)	145 ± 19^a^	134 ± 30^ab^	145 ± 22^a^	123 ± 17^b^	101 ± 27^c^
Peri-uterine fat (g)	15.5 ± 2.2^a^	13.6 ± 1.5^ab^	16.0 ± 1.9^a^	12.1 ± 1.7^b^	9.9 ± 1.8^c^
Retroperitoneum fat (g)	11.3 ± 1.6^a^	9.7 ± 1.1^b^	11.7 ± 1.6^a^	8.2 ± 0.9^c^	6.0 ± 0.8^d^
Visceral fat mass (g)	26.8 ± 3.1^a^	23.3 ± 2.9^b^	27.7 ± 2.9^a^	20.4 ± 2.6^c^	15.9 ± 2.3^d^
Food intake (g/day)	13.9 ± 1.7	14.2 ± 1.7	14.5 ± 1.8	14.0 ± 1.3	13.2 ± 1.6
Energy expenditure (kcal/kg^0.75^/day)	89.4 ± 10.2^b^	94.0 ± 10.7^b^	88.6 ± 9.6^b^	105.5 ± 11.5^a^	110.1 ± 12.7^a^
Carbohydrate oxidation (mg/kg^0.75^/min)	5.1 ± 0.7	5.3 ± 0.7	5.0 ± 0.7	4.9 ± 0.7	5.3 ± 0.8
Fat oxidation (mg/kg^0.75^/min)	4.4 ± 0.6^b^	4.7 ± 0.6^b^	4.4 ± 0.7^b^	6.3 ± 0.7^a^	6.4 ± 0.8^a^

Visceral fat was the sum of epididymal fat pads and inguinal fat. Values represented means ± standard deviation (*n* = 10)

Different letters represent a significant difference in Tukey test at *p* < 0.05

*CFM* 1% water extract of cauliflower mushroom, *LFE* 0.1% *L. fermentum*, *CFLF* 1% water extract of cauliflower mushroom +0.1% *L*. *fermentum*, *positive-control* 30 μg/kg body weight 17β-estradiol

### Body composition

**Fig. 1:**
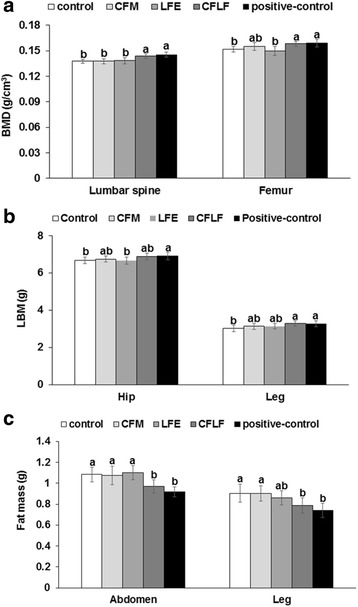
Body composition including bone mineral density (BMD), lean mass (LM), and fat mass (FM) measured by DEXA. Control, OVX rats fed a high-fat diet (HFD) with 1% dextrin; CFM, OVX rats fed an HFD with 1% lyophilized cauliflower mushroom; LFE, OVX rats fed an HFD with 0.1% *L*. *fermentum* plus 1% dextrin; CFLF, OVX rats fed an HFD with 1% CFM plus 0.1% *L*. *fermentum*; positive-control, OVX rats fed an HFD with 1% dextrin plus 30 μg/kg body weight 17β-estradiol. At the end of the experimental period, BMD (**a**) in the lumbar spine and femur and LM (**b**) and FM (**c**) in the abdominal and leg regions were measured by DEXA. Values are expressed as means ± SD (*n* = 12). Different letters represent a significant difference in Tukey test at *p* < 0.05

BMD in the lumber spine and femur was much lower in the OVX-control group than the positive-control group (Fig. 1a). CFLF prevented the decrease in BMD in the lumber spine and femur as much as the positive-control although neither CMF nor LFE prevented it (Fig. 1a). LBM in the hip and leg was also lower in the OVX-control group than the normal-control group, and CFLF exhibited a similar LBM as the positive-control group (Fig. 1b). In contrast to the LBM, fat mass in the abdomen and leg was much higher in the OVX-control group than the positive-control (Fig. 1c). CFLF lowered fat mass in the abdomen and leg as much as the positive-control group.

### Lipid metabolism

OVX-control rats exhibited dyslipidemia including elevated serum total cholesterol, LDL cholesterol, and triglyceride concentrations and lower serum HDL cholesterol concentrations in overnight-fasted states in comparison to the normal-control rats (Table 3). CFM prevented the decrease of serum HDL cholesterol and triglyceride concentrations but not total and LDL cholesterol concentrations in the circulation (Table 3). CFLF protected against the development of dyslipidemia in OVX rats, and lipid profiles in the blood were similar to the positive-control group.

**Table 3:** Glucose and lipid metabolism in overnight-fasted states

	Control	CFM	LFE	CFLF	Positive-control
Total cholesterol (mg/dL)	146.4 ± 16.4^a^	149.1 ± 15.2^a^	147.4 ± 15.2^a^	126.3 ± 13.0^b^	123.2 ± 12.5^b^
HDL cholesterol (mg/dL)	17.7 ± 1.8^c^	24.8 ± 2.6^a^	19.9 ± 2.2^c^	24.1 ± 2.5^a^	20.9 ± 2.2^b^
LDL cholesterol (mg/dL)	106.9 ± 11.0^a^	107.3 ± 12.0^a^	106.1 ± 11.0^a^	84.8 ± 8.8^b^	86.4 ± 9.1^b^
Triglyceride (mg/dL)	108.8 ± 12.2^a^	85.4 ± 8.9^bc^	107.1 ± 12.0^a^	87.1 ± 9.1^b^	79.4 ± 8.3^c^
Serum glucose (mg/dL)	122.2 ± 10.7^a^	115.8 ± 8.4^ab^	116.6 ± 7.7^ab^	113.7 ± 8.6^b^	112.9 ± 9.1^b^
Serum insulin (ng/mL)	2.87 ± 0.42^a^	2.05 ± 0.31^b^	1.81 ± 0.34^c^	1.77 ± 0.26^c^	1.96 ± 0.34^bc^
HOMA-IR	8.4 ± 1.2^a^	5.9 ± 0.9^b^	5.2 ± 0.8^b^	4.5 ± 0.6^c^	5.5 ± 0.7^b^
HOMA-B	367 ± 44^a^	280 ± 40^b^	256 ± 31^b^	227 ± 28^c^	283 ± 32^b^

Values represented means ± standard deviation (*n* = 10)

Different letters represent a significant difference in Tukey test at *p* < 0.05

*CFM* 1% water extract of cauliflower mushroom, *LFE* 0.1% *L*. *fermentum*, *CFLF* 1% water extract of cauliflower mushroom +0.1% *L*. *fermentum*, *positive-control* 30 μg/kg body weight 17β-estradiol

### Glucose metabolism

Serum glucose concentrations in overnight-fasted states were higher in the OVX-control rats than the normal-control rats, and CFLF lowered it to concentrations as low as the positive-control rats. Serum insulin concentrations were much higher in the OVX-control group than the positive-control group (Table 3). Serum insulin concentrations were lower in the descending order of OVX-control, CFM, positive-control, LFE, and CFLF. Consistent with serum insulin concentrations, HOMA-IR, an index of insulin resistance, was much higher in OVX-control rats than the positive-control. HOMA-IR was lower in CFM and LFE than in OVX-control, and it was lowest in CFLF among all the groups (Table 3).

**Fig. 2:**
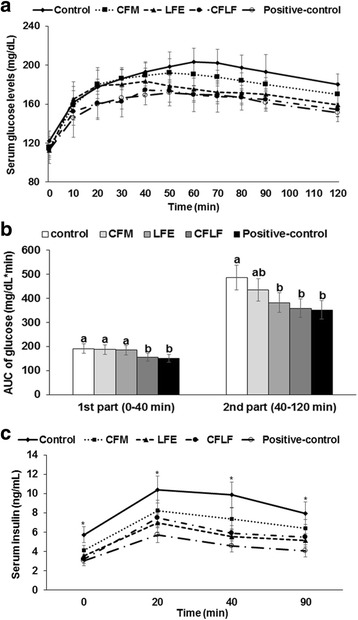
Serum glucose concentrations and area under the curve of glucose and insulin during oral glucose tolerance test (OGTT). Control, OVX rats fed a high-fat diet (HFD) with 1% dextrin; CFM, OVX rats fed an HFD with 1% lyophilized cauliflower mushroom; LFE, OVX rats fed an HFD with 0.1% *L*. *fermentum* plus 1% dextrin; CFLF, OVX rats fed an HFD with 1% CFM plus 0.1% *L*. *fermentum*; positive-control, OVX rats fed an HFD with 1% dextrin plus 30 μg/kg body weight 17β-estradiol. Changes of serum glucose concentration were measured during OGTT (**a**) and the average of the area under the curve (AUC) of glucose during the first part (0–40 min) and second part (40–120 min) of OGTT (**b**) was given. The changes of serum insulin levels were shown (**c**). Each dot and bar represents the mean ± SD (*n* = 12). Asterisk indicates significantly different among the treatments of OVX rats at *p* < 0.05. Different letters represent a significant difference in Tukey test at *p* < 0.05

OVX-control rats had higher serum glucose concentrations that continued to increase until 60 min after glucose challenge, and then slowly decreased in comparison to the positive-control rats, indicating that the OVX-control rats had increased insulin resistance (Fig. 2a). CFM and LFE rats had peak of serum glucose concentrations at about 40 min, and the concentrations at 40 min were similar in the OVX-control (Fig. 2a). However, serum glucose in CFM and LFM quickly decreased after the peak levels in comparison to the OVX-control group. The peak concentrations in the CFLF and positive-control rats were lower than the OVX-control group, and the levels quickly declined Fig. 2a). Since glucose concentration of most rats peaked at 40 min, the AUC of serum glucose levels was divided into two parts at 40 min after glucose loading. The first part of AUC represented the glucose metabolism by insulin secretion capacity and insulin sensitivity at hyperglycemia whereas the second part of AUC was mainly explained by insulin sensitivity (Fig. 2b). Serum insulin concentrations during OGTT were much higher in control rats than in positive-control rats whereas the levels were lower in CFM, LFE, and CFLF than the control (Fig. 2c). However, the decrease by the treatments was not as great as the positive-control. The AUC of serum insulin levels was much higher in the control group than in the positive-control group, whereas it was lower in descending order of the control (630 ± 93 ng/mL min), CFM (443 ± 64 ng/mL min), CFLF (344 ± 49 ng/mL min), LFE (316 ± 43 ng/mL min), and positive-control (227 ± 34 ng/mL min).

**Fig. 3:**
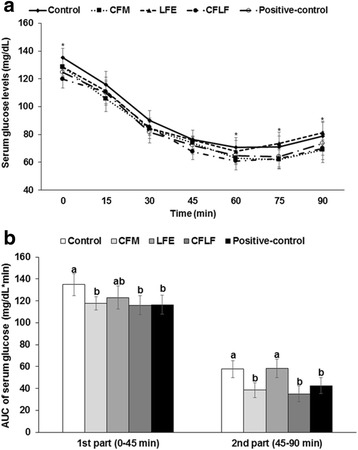
Changes in serum glucose concentrations during the insulin tolerance test (ITT). Control, OVX rats fed a high-fat diet (HFD) with 1% dextrin; CFM, OVX rats fed an HFD with 1% lyophilized cauliflower mushroom; LFE, OVX rats fed an HFD with 0.1% *L*. *fermentum* plus 1% dextrin; CFLF, OVX rats fed an HFD with 1% CFM plus 0.1% *L*. *fermentum*; positive-control, OVX rats fed an HFD with 1% dextrin plus 30 μg/kg body weight 17β-estradiol. ITT was conducted with intraperitoneal injection of 0.75 IU insulin/kg body weight and measured serum glucose concentrations in blood collected from the tail every 15 min for 90 min. Changes of serum glucose concentrations were shown during ITT (**a**). The average of the area under the curve (AUC) of glucose during the first part (0–45 min) and second part (45–90 min) of ITT (**b**) was given. Each dot and bar represents the mean ± SD (*n* = 12). Asterisk indicates significantly different among the treatments of OVX rats at *p* < 0.05. Different letters represent a significant difference in Tukey test at *p* < 0.05

In IPITT, serum glucose levels markedly decreased until 30–45 min, and then began to rebound from 60 min in all rats after an intraperitoneal injection of insulin (Fig. 3a). After no food for 6 h, serum glucose levels were higher in OVX-control rats than in the positive-control rats and CFLF also lowered the serum glucose levels in OVX rats (Fig 3a). AUC of serum glucose concentrations during the first part and second part was higher in OVX-control group than in positive-control group whereas CFM and CFLF lowered the AUC as much as the positive-control group (Fig. 3b). OVX-control rats had lower insulin sensitivity and 17β-estradiol, but CFM and CFLF improved the insulin sensitivity.

### Hepatic insulin signaling

**Fig. 4:**
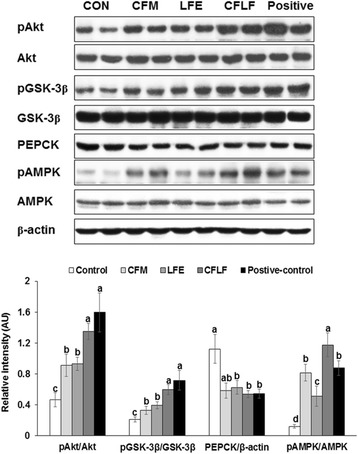
Hepatic insulin signaling. Control, OVX rats fed a high-fat diet (HFD) with 1% dextrin; CFM, OVX rats fed an HFD with 1% lyophilized cauliflower mushroom; LFE, OVX rats fed an HFD with 0.1% *L*. *fermentum* plus 1% dextrin; CFLF, OVX rats fed an HFD with 1% CFM plus 0.1% *L*. *fermentum*; positive-control, OVX rats fed an HFD with 1% dextrin plus 30 μg/kg body weight 17β-estradiol. Hepatic insulin signaling was determined by immunoblotting assays. Each bar represents the mean ± SD (*n* = 4). Different letters represent a significant difference in Tukey test at *p* < 0.05

The phosphorylation of Akt and GSK-3β were much lower in the control group than in the positive-control, and it increased in the ascending order of control, CFM, LFE, CFLF, and positive-control (Fig. 4). PEPCK expression was higher in the control than in the positive-control and CFM, LFE and CFLF decreased its expression. The phosphorylation of AMPK was much lower in the control than in the positive-control, and CFM and CFLF prevented the decrease (Fig. 4). The phosphorylation of AMPK was higher in CFLF than in the positive-control.

### Gut microbiome

**Fig. 5:**
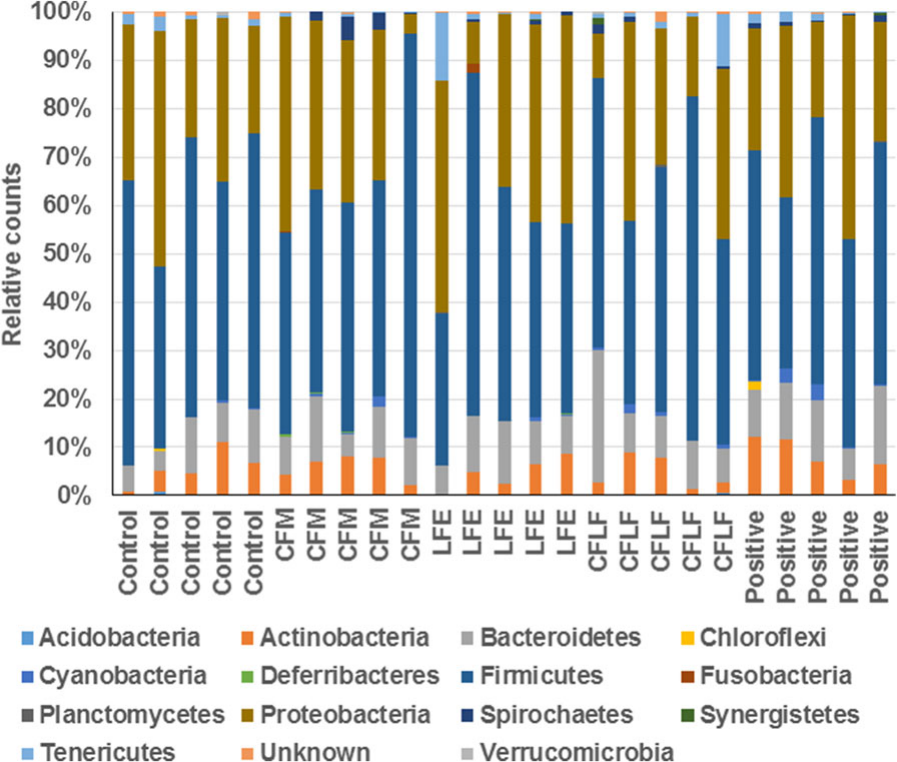
Proportion of taxonomic assignments [Phylum] for gut microbiomes. Control, OVX rats fed a high-fat diet (HFD) with 1% dextrin; CFM, OVX rats fed an HFD with 1% lyophilized cauliflower mushroom; LFE, OVX rats fed an HFD with 0.1% *L*. *fermentum* plus 1% dextrin; CFLF, OVX rats fed an HFD with 1% CFM plus 0.1% *L*. *fermentum*; positive-control, OVX rats fed an HFD with 1% dextrin plus 30 μg/kg body weight 17β-estradiol

OVX rats exhibited different proportions of taxonomic assignment (Fig. 5). OVX increased the ratio of *Firmicutes* to *Bacteroidetes*, and the changes were reversed in the positive-control group (Table 4). CFM and LFE prevented the increase in the ratio of *Firmicutes* to *Bacteroidetes*, but it was not as much as CFLF. CFLF lowered the ratio to similar values as the positive-control (Table 4). The number of proteobacteria was reduced in the CFM and CFLF groups, compared to the control group, but it was not significantly different between the control and positive-control groups (Table 4). The growth of proteobacteria was not affected by estrogen deficiency, but it was modulated by β-gluten.

**Table 4:** Gut microbiomes (unit: counts)

	Control	CFM	LFE	CFLF	Positive-control
Actinobacteria	199 ± 31^b^	209 ± 36^b^	142 ± 29^c^	161 ± 28^c^	295 ± 42^a^
Firmicutes	1837 ± 241^a^	1548 ± 224^b^	1427 ± 251^b^	1593 ± 232^b^	1549 ± 236^b^
Proteobacteria	1323 ± 186^a^	960 ± 167^b^	1232 ± 184^a^	928 ± 142^b^	1365 ± 192^a^
Bacteroidetes	268 ± 44^c^	275 ± 36^c^	295 ± 42^c^	368 ± 48^b^	421 ± 51^a^
Cyanobacteria	5.8 ± 1.1^d^	19.4 ± 4.7^c^	7.2 ± 1.7^d^	28.8 ± 6.6^b^	54.6 ± 12.2^a^
Ratio of F/B^1^	6.85 ± 1.01^a^	5.64 ± 0.87^b^	4.84 ± 0.79^bc^	4.33 ± 0.64^c^	3.68 ± 0.61^c^

Values represented means ± standard deviation (*n* = 5)

Different letters represent a significant difference in Tukey test at *p* < 0.05

*CFM* 1% water extract of cauliflower mushroom, *LFE* 0.1% *L*. *fermentum*, *CFLF* 1% water extract of cauliflower mushroom +0.1% *L*. *fermentum*, *positive-control* 30 μg/kg body weight 17β-estradiol

^1^The ratio of Firmicutes and Bacteroidetes

## Discussion

Gut microbiota has a profound influence on metabolism, immunity, and protection against pathogens. The gut microbiota also influences obesity and BMD [17, 30], and probiotics, prebiotics, and synbiotics may attenuate menopausal symptoms by modulating gut microbiota in estrogen-deficient animals. However, the efficacy of probiotics, prebiotics, and synbiotics for improving gut microbiota still remains controversial. The present study suggested that OVX changes gut microbiota and the changes are associated with menopausal symptoms, but that the synbiotics, CFM and LFE, can prevent many of the gut microbiota changes and menopausal symptoms in estrogen-deficient animals. CFM itself also alleviated the metabolic disturbances of energy, glucose, lipid, and bone metabolism in OVX rats but it was not as much as CFLF. LFE itself had the least effect. Thus, the synbiotics might be effective for alleviating menopausal symptoms.

Cauliflower mushrooms mainly contain six-branched 1,3-β-glucan (about 40% of the mushroom weight) and small amounts of the flavor components including 3-octanone, DL-3-octanol, and 1-octen-3-ol and vitamin D_2_ [31]. Oat β-glucans have been mainly known to lower blood glucose and cholesterol levels in animals and humans [32, 33]. However, cauliflower mushroom has been shown to possess anti-bacterial and anti-fungal, anti-inflammatory hypocholesterolemic, and immunomodulatory activities [18, 29, 31]. Thus, β-glucans of different origins might have similar or different activities against metabolic disturbances. β-Glucans in cauliflower mushroom, six-branched 1,3-beta-glucan, are different from β-glucans in oats and barleys. However, no studies have investigated CFM effects on menopausal symptoms. The present study showed some beneficial synbiotic effects of CFM and LFE in an estrogen-deficient animal model.

Probiotics and prebiotics can change gut microbiota, but neither of them consistently changes gut microbiota efficiently and synbiotics, a mixture of probiotics and prebiotics, may be more effective for making beneficial changes to the gut microbiota [34]. Probiotics, prebiotics, and synbiotics have mainly been reported to improve bowel-related diseases such as irritable bowel syndrome and colon cancer [35]. In addition, a “leaky gut” may deliver toxins, antigens, or bacteria into the liver and may play a pathogenic role in liver damage [36]. In this aspect, gut microbiota may play an important role by improving intestinal barriers. Recently, gut microbiota have been associated with obesity and its related diseases such as type 2 diabetes and dyslipidemia.

*Lactobacillus* species have a common characteristic of producing lactic acid or alcohol from sugars. *Lactobacillus* species have probiotic activities, but different species have specific activities. *L*. *fermentum* produces lactic acid from sugars, and it is found in sourdough. Since *L*. *fermentum* tolerates acid environments and 3 g/L of bile salts, it is found to survive in the intestines and it can act as a probiotic, although *L*. *fermentum* does not use dietary fiber [37]. *L*. *fermentum* is safe up to a level of 10^10^ CFU/kg body weight/day during 2–4 weeks of treatment in animals, and it has also been shown to be safe in infant formulas in 1–6-month old infants [38, 39]. It enables harmful intestinal bacterial enzymes to be inhibited, decreases pathogenic bacterial populations, and increases beneficial bacterial populations [38]. Interestingly, *L*. *fermentum* was first isolated from healthy elderly Koreans [40] and it has the strongest binding to intestinal epithelial cells and has potent immune-enhancing, anti-inflammatory, anti-oxidative, and anti-dyslipidemia activities and reverses alcohol-induced liver diseases [14, 41, 42]. These studies suggest that *L*. *fermentum* is a beneficial probiotic in humans. However, it has not been studied for alleviating menopausal symptoms. Previous studies have demonstrated that *L*. *reuteri* and *Bifidobacterium longum* increased BMD in OVX rats [30, 43] whereas in the present study, LFE itself did not change BMD, but CFM + LFE elevated BMD to densities as high as the positive-control group. LFE improved glucose tolerance and insulin resistance compared to the control group. In other menopausal symptoms, LFE itself had limited capacity for improving menopausal symptoms in OVX rats, but LFE plus CFM prevented menopausal symptoms as much as 17β-estradiol treatment in the present study. These results suggested that LFE itself did not improve menopausal symptoms as much as CFLF and positive-control groups, but it was associated with the changes of gut microbiota flora. Thus, symbiotic treatment of CFLF may be beneficial for post-menopausal women.

CFM has been reported to have immunomodulatory, anti-tumor, and hypocholesterolemic activities in animals [18, 29, 31], and also β-glucans, the major components of CFM, have hypoglycemic activity [44]. *Bacteroidetes* are well-known bacteria that use dietary fibers as an energy source, and their growth may be increased with CFM [45]. Thus, *Bacteroidetes* can be increased in the intestines when CFM is provided to the animals. CFM may improve the estrogen deficiency-linked disturbances of energy, glucose, lipid, and bone metabolism by modulating gut microbiota. The present study showed that CFM reduced visceral fat mass, serum triglyceride levels, and insulin resistance. CFM partly reduced the ratio of *Firmicutes* and *Bacteroidetes* in comparison to the control group. However, CFLF improved menopausal symptoms much better than CFM. Therefore, CFLF, but not CFM, was sufficient for protecting against menopausal symptoms.

Early studies of the gut microbiomes concentrated on selectively stimulating the growth of a limited number of health-promoting bacteria; the application of prebiotics was focused on improving host welfare [10]. However, the applications of synbiotics have recently progressed to the alleviation of metabolic diseases [34]. Some studies have reported that synbiotics have beneficial effects on obesity and metabolic syndrome in human clinical studies [46] and synbiotics are somewhat better than individual effects on conditions such as obesity, ethanol-induced hepatic steatosis, and inflammation-related diseases [34, 47]. The present study also definitively demonstrated that synbiotics had much better efficacy for improving menopausal symptoms than either CFM or LFE alone. CFLF prevented the disturbance of energy, glucose, and lipid metabolism in OVX rats and also it reduced fat mass and increased BMD. In parallel with the alleviation of menopausal symptoms, the gut microbiota in the CFLF group showed similar patterns as the positive-control group: CFLF reduced the ratio of *Firmicutes* and *Bacteroidetes* and increased total counts of bacteria in the gut.

## Conclusions

Estrogen deficiency causes menopausal symptoms including higher tail skin temperature, increasing visceral fat mass, dyslipidemia, glucose intolerance, and BMD loss. Additionally, estrogen-deficient rats had higher ratios of *Firmicutes* to *Bacteroidetes* and lower total bacterial counts. CFM containing mainly β-glucans partly prevented the disturbance of glucose and lipid metabolism while potentiating hepatic insulin signaling without changing serum 17β-estradiol levels. Although LFE itself did not protect against the metabolic disturbance caused by estrogen deficiency, CFLF synergistically improved energy, glucose, lipid, and bone metabolism as much as the positive-control group. The improvement of metabolism was associated with the prevention of gut microbiota patterns. Therefore, the synbiotics, CFM and LFE, can be a potential therapeutic agent for menopausal symptoms in post-menopausal women.

## Abbreviations

AMPK: AMP kinase
AUCG: Area under the curve of glucose
AUCI: Area under the curve of insulin
BMD: Bone mineral density
CFLF: 1% CFM plus 0.1% LFE
CFM: Cauliflower mushroom plus 0.1% casein
DEXA: Dual-energy X-ray absorptiometer
En%: Energy percent
ER: Estrogen receptor
GSK-3β: Glycogen synthase-3β
HOMA-IR: Homeostasis model assessment estimate of insulin resistance
IL-1β: Interleukin-1β
IPITT: Intraperitoneal insulin tolerance test
LFE: 0.1% *L*. *fermentum* plus 1% dextrin
MCP-1: Monocyte chemoattractant protein-1
OGTT: Oral glucose tolerance test
OVX: Ovariectomy
PEPCK: Phosphoenol-pyruvate carboxykinase
PKB/Akt: Protein kinase B
TNF-α: Tumor necrosis factor-α
VCO_2_: Average carbon dioxide production
VO_2_: Average oxygen consumption
